# The science of talk in clinical science: using a conversation analytic approach as a foundation for communication skills training

**DOI:** 10.15694/mep.2019.000224.1

**Published:** 2019-12-09

**Authors:** Sarah J White

**Affiliations:** 1 Macquarie University

**Keywords:** communication skills, pre-clinical, undergraduate, conversation analysis, clinical science, professional practice, core skills

## Abstract

This article was migrated. The article was marked as recommended.

Communication skills are considered a central part of medical and health professional curricula. The focus for both theoretical knowledge and practical skills in these curricula is often, necessarily, on that which is directly relevant to consultations and other clinical activities. Prior to engaging in this more specific and often experiential learning, it is arguable that the inclusion of foundational learning around how interaction works to adequately scaffold more specific, clinically-contextualised learning, building through the zone of proximal development. In this paper, I describe a conversation analysis-informed curriculum for communication skills in an undergraduate pre-clinical science degree which is designed to enhance the ability to critically and constructively reflect on their own communication.

## Introduction

Clinical communication skills are taught in a variety of ways at all levels of training ( Kurtz *et al*., 2003; Stewart *et al*., 2013; Silverman, Kurtz and Draper, 2013) and form an important part of medical and health professional curricula. This teaching involves varying degrees of input from communication and language sciences ( Bachmann *et al*., 2013; Silverman, Kurtz and Draper, 2013), often with a focus on theory specifically related to clinical communication and applying these skills in a context through experiential learning activities. Such training is valuable ( Rotthoff *et al*., 2011), however there are ways to improve the scaffolding of such learning, as described by Street and De Haes (2013), who argue for the inclusion for a theoretical framework within such curricula. This is particularly relevant to undergraduate students for whom learning more foundational aspects of interaction may be closer to their actual level of development ( Podolskiy, 2012). With that in mind, and in the context of a broader core stream on professional practice, we integrated a foundational communication curriculum into a pre-clinical program. While conversation analysis (CA) and other forms of interaction analysis have been used in communication training within clinical professions and in other industries ( Antaki, 2011; Stokoe, 2011), the building of a foundational, CA-informed curriculum for communication skills in the pre-clinical years is yet to be described in the literature. The purpose of this paper is to describe this curriculum, providing a rationale for including interaction theory in the introductory phases on clinical communication training.

Communication skills training involves introducing students to the skills for effectively communicating in consultations, with colleagues, and beyond ( Bachmann *et al*., 2013; Makoul, 2001; Noble *et al.*, 2018). In the recent Consensus statement on an updated core communication curriculum for UK undergraduate medical education, the authors maintain that “an appreciation of conceptual frameworks and research evidence” ( Noble *et al.*, 2018, p. 1715) is at the core of communication curriculum. This approach, however, focuses on frameworks and evidence relating specifically to communication in clinical settings and does not explicitly suggest that scaffolding learning with more introductory information on communication is also of merit.

Communication, more specifically interaction, is how we get things done together in the world ( Heritage and Clayman, 2010). It is how we order coffee, deliver a lecture, have yet-another-meeting; it is a way to display our culture and our identity; and it is how clinicians weave through a history taking to formulate a diagnosis, handover a patient to the next person on, and work as a team in an operating theatre. Interaction, in all its parts and its manifestations, makes society possible. These assumptions about how we participate in this social world through interaction, how we make sense of each other and build conversations together, are part of the theoretical framework that underpin conversation analysis. Developing an understanding of how interaction works as a basis for social life is key to informing the appreciation of the frameworks and evidence that ultimately form the basis of clinical communication skills training.

In this article, I describe the learning activities and assessments within the communication component of the curriculum, demonstrating the constructive alignment in its design. I also briefly comment on future plans for the curriculum.

## What we did

In a pre-clinical undergraduate degree, the Bachelor of Clinical Science, at Macquarie University, we include a series of units focussed on professional practice. These units are designed to make explicit the core skills for students pursuing careers in health and medicine, whether as clinicians, researchers, or other professionals. This includes communication. In developing the communication curriculum, the aim was to create a cohesive and authentic journey from the basics of how interaction works to introducing students to effective communication in clinical settings. As there is a lot of ground to cover on these topics, ensuring the fundamentals were covered in a clear but appropriately contextualised way, was a key focus of curriculum design.

These units generally follow a flipped model, with students preparing with readings and recorded talks prior to a two-hour tutorial. Across the professional practice units, communication is also covered within the context of both teamwork and broader society, exploring culture, gender, and privilege in the latter. Communication is explicitly covered in seven weeks (out of 39 weeks of professional practice units) under the following topics:

### Block 1 - first year level unit

• Communication as action
• Nonverbal communication
• Analysing communication

### Block 2 - third year level unit

• Communication in the workplace
• Managing uncertainty in communication
• Clinical communication
• Clinical handover

In the most recent iteration of the degree, the first unit is core while the second is elective, so students have a minimum of three weeks of explicit communication learning. The following is a brief description of the preparation work and in-class activities for each of the seven weeks.

### Communication as action

#### Learning objectives: Define core concepts of communication; Identify key skills required for effective communication

The first three weeks on communication occur early within the degree and are about foundations of understanding and analysing communication. In this first week, the focus is engaging students in describing communication as a social enterprise - as how we get things done, not just in health and medicine, but in everyday life. In the preparation work, students watch two brief in-house videos on communication - one introducing the theme overall (5 minutes) and one introducing the concept of norms in interaction (16 mins), specifically the structural and sequential organisation of talk ( Heritage and Clayman, 2010). The students read the chapter on language use in Clark’s *Using Language* ( 1996). This introduces students to the concept of language and interaction as joint action - how we get things done together. Following these, students also prepare an example of communication in everyday activities, by selecting an activity such as going to the movies and listing the different communication activities involved in doing that activity. In the tutorial, the students work in groups to identify the different communication skills (e.g. listening, asking questions, responding) required in these tasks. Students then rank these in terms of generalisability across the different activity types, to discover which are the most common. Each student then picks one of these skills and is asked to briefly describe why it would be important in clinical communication.

Flowing on from this are two weeks that are swapped depending on timing requirements (one is usually an online week due to the scheduling of the unit). Here they are presented in the preferred order.

### Nonverbal communication

#### Learning objective: Describe the role of nonverbal communication in interaction

This is an online week and one that uses sources beyond conversation analysis and sociolinguistics. As it is online, the work involves preparation and an online activity. The preparation materials are a short in-house video introducing the topic (4 mins), an online tutorial via sophia.org (Sophia Tutorial, 2019), a reading on the evolutionary basis of nonverbal communication ( Frank and Shaw, 2016), and an activity where students are asked to view a Charlie Chaplin video (2007) and take note of non-verbal communication. For online delivery, there is a forum where students are asked to share their thoughts on the preparation work. When delivered face-to-face, this was moved into an open in-class discussion with the addition of another activity where students work in small groups to choose one non-verbal behaviour and describe how its meaning changed within different interactional contexts. This additional activity was helpful in emphasising the importance of interactional context for understanding nonverbal communication.

### Analysing communication

#### Learning objectives: Explain why using analytic tools can help improve communication; Describe how communication changes based on its purpose

The focus of this week is firmly back within the CA tradition, with two videos relating to analysing conversation to introduce the online materials (one in-house, 11 mins; one TED Talk by Elizabeth Stokoe (2014), 19 mins). Following this, students listen to a half-hour podcast on turn-taking ( Rosen and Wright, 2016) and read a Chapter 3 in Talk in Action on CA theory ( Heritage and Clayman, 2010). These pre-class activities are designed to provide more depth to the initial two introductory weeks and to move students from simply learning about interaction to learning that it can be analysed. In class, students participate in small group and large group facilitated discussions on the preparation materials and apply their understanding of communication activities and skills to different scenarios, such as the courtroom. They also analyse an audio-recording of a short clip of a clinical interaction.

For assessment preparation, which occurs at the end of these three weeks, students also complete section 1 and 2 of an external online tutorial ( Llewellyn, 2019). Assessment is described in more detail below.

### Communication in the workplace

#### Learning objective: Describe similarities and differences between social and workplace communication activities

There is almost a year between the first three weeks and the next four on communication, however, as mentioned above, other related topics such as teamwork and culture are explored during that time. In this block, the focus is on contextualising what has previously covered, with a specific focus on the clinical work environment.

The first week focuses on workplace communication, drawing from sources in CA and beyond. The students prepare by watching three 10-minute in-house video lectures on workplace communication, covering an introduction, pragmatics, and rapport. This is coupled with a reading about institutional talk within a CA-framework ( Heritage and Clayman, 2010 Ch. 4). Due to scheduling requirements, this is an online week, so students complete a quiz based on the preparation material and then participate in a forum where they discuss the concept of rapport as this is an often requested but rarely explained clinical communication skill.

### Managing uncertainty in communication

#### Learning objectives: Explain your personal, evidence-informed approach to learning clinical communication; Demonstrate flexibility in uncertain communication scenarios

This week involves two aspects, as indicated in the learning objectives. Prior to class the students read an article that challenges the established conceptualisation of communication skills ( Salmon and Young, 2011). They then search for a published response to that article (e.g. Lefroy and McKinley, 2011; Silverman *et al.*, 2011) and post their own view on these approaches to clinical communication training. This engages them in critical thinking about communication theory and in developing their own evidence-informed framework.

The face-to-face activities here are somewhat different, with a drama teacher, who has experience as a simulated patient and as an improvisation trainer, facilitating a two-hour improvisation workshop which also includes a brief patient-clinician role play at the end. This kind of learning activity has been used successfully for clinical communication training ( Hoffman, Utley and Ciccarone, 2008; Terregino *et al.*, 2019; Watson and Fu, 2016) and, for these students, is helpful in enabling them to approach role-play confidently. As role play is a frequently used communication training tool in clinical degree programs, this is an important skill to develop.

### Clinical communication

#### Learning objectives: Identify the key skills in clinical communication; Describe the implications of question design on responses particularly in clinical settings

Building on the confidence developed in the improvisation workshop, this week focuses on applying the theoretical knowledge in a more experiential setting. The preparation work is focused on two readings, one of which covers consultation skills ( Silverman, Kurtz and Draper, 2013 Ch. 1) and the other which delves into the conversation analytic evidence on history taking in medicine ( Heritage and Clayman, 2010, Ch. 10). Using these chapters, the students participate in an online discussion forum where they choose an evidence-based motivation for improving clinical communication and briefly describe why it is important to them, further developing their own approach to communication. This also draws the learning back to the fundamental principle of appreciating the importance of effective clinical communication ( Noble *et al.*, 2018).

In class, students engage in a large group discussion and an analysis of a consultation excerpt, using audio and a transcript from 30-second clip of a doctor-patient consultation, where they identify process, content, and perceptual skills ( Silverman, Kurtz and Draper, 2013). We then move to small group role plays where students practice introducing themselves as a clinical student. This is done in groups of three, where each student has a turn at being the patient, the clinician, and the observer ( International Association for Communication in Healthcare, 2010). This is followed by a whole class observation of a role play by some of the students in a scenario where they are patients being collected from the waiting room. We ask some of the students role playing as patients to add challenges, such as being distracted on their phone, or being grumpy due to the doctor running late. The class is wrapped up with a small group activity where the students work to identify how small changes to a description of a patient in a history taking scenario would alter their approach, with reference to their preparation material. This debrief about the application of theoretical knowledge, particularly relating to interactional structures and practices, is then extended to understanding their participation within the role play paradigm.

### Clinical handover

#### Learning objective: Synthesise aspects of communication and teamwork to explain clinical handover in the context of safety and quality in healthcare

This final week is not only the final week on communication but in this unit of study. It is designed as a capstone to assist students in drawing together the different threads covered across the semester. As such the preparation work reaches beyond CA and communication and includes a 20 minute TEDTalk on collective competence ( Lingard, 2013), a short article on clinical handover ( Jorm, White and Kaneen, 2009), another on team competence ( Lingard, 2016), and information from the Australian Commission on Safety and Quality in Healthcare ( Australian Commission on Safety and Quality in Health Care, 2019). This is supported by a quiz which is specifically designed to highlight the key points across these resources.

Class starts with a medium-sized group activity of the telephone game. Instead of a clinical scenario, we return to the everyday. This time students are role-playing working in a café where they are required to handover a very complex coffee order and seating arrangement for a large group. Two students act as observers, taking notes about the inconsistencies that appear. The activity is followed by a large group debrief, in which we discuss how and why these inconsistencies occurred, drawing on learning from across the semester. This is expanded into a more general wrapping up of the semester.

### Assessment

Central to this process was designing assessments that were authentic and that were constructively aligned. This was achieved through a continuation of the situated cognition approach ( Ataizi, 2012) as assessments were designed to assist students in both developing their knowledge and skills across time as well as engaging with the real-life application of what they were learning. There are two assessments specifically relating to communication, although other assessments, such as written and presentation tasks, also have assessable components on communication. The first is early in the course and is an in-class activity where students view a one-minute clip of a recorded clinical consultation and write commentary on aspects of verbal and non-verbal communication. This assessment is designed to engage students in applying the concepts they have learned in the previous three weeks while also exposing them to the complexity of naturally-occurring interaction.

The second is during the second block of communication weeks and is an activity where students record themselves doing a consultation role-play with a fellow student. They choose one minute of this to transcribe and then analyse it, relating their reflection to the literature. This kind of task utilised in other fields, such as English language teaching ( Lynch, 2001), and is useful in encouraging students to critically reflect on their own communicative practices in a constructive way.

These assessments have been successful in assisting students in identifying their own development as students of communication and as communicators. By creating assessments that clearly progress in difficulty and application as well as ones that are practical in their approach, students are guided to see the benefit of not only the assessments themselves but the learning activities that have been structured to support them.

## What’s next?

As a relatively new degree, the Bachelor of Clinical Sciences has been evaluated for quality improvement purposes and a variety of modifications have been made. Now that the communication curriculum is more settled, a targeted evaluation on that specific aspect would be appropriate. This would ideally capture the applicability of learning for use within the degree as well as beyond the degree in postgraduate study and work.

Following on from these units, students also have access to an online *Connected Curriculum* module on the clinical consultation. The *Connected Curriculum* is a series of evidence-based online modules across a wide range of topics within health and medicine that are available to students and staff Faculty-wide. They are often embedded in the curriculum and shared through the primary eLearning platform, with students being directed to specific modules for pre- and post-classroom learning or as standalone activities. The clinical consultation module is based on a unit offered in previous iteration of the degree which specifically concentrated on communication in the clinical consultation, which, while well-received by students, particularly who continued on to study medicine, was too focused on application more relevant to a clinical degree. This revision of the curriculum suggests that the seven weeks of communication-specific learning is an important foundation for further study and in the workplace.

As the *Connected Curriculum* becomes a more integrated part of the degrees available in the Faculty of Medicine and Health Sciences, it is anticipated that some of the resources available in it will be integrated into the online learning activities within the pre-clinical communication curriculum and that the delivery of that curriculum will also inform the ongoing development of further online communication modules for integration into other degrees.

## Conclusions

Establishing a solid foundational understanding of how interaction works is achieved through the use of a well-evidenced theoretical approach. It can be integrated to scaffold student learning within communication generally while also looking to future clinical communication learning. Clinical communication is taught by a range of people in the classroom, on placement, and in the workplace. The people teaching bring a range of important perspectives and experience, however it is fraught with difficulty as the interest, engagement, and expertise of tutors and supervisors varies markedly. Building an understanding of the science of talk ( Albert *et al.*, 2018) serves to give students the confidence in their interpretation of their learning as a student observing, role-playing, and engaging in activities requiring effective clinical communication.

Beyond an appreciation for the clinical implications of effective communication, this approach emphasises the necessity of communication in every part of social life. It encourages students to engage with more than a list of skills to master in order to communicate well and instils an appreciation of the science of talk and the complexity of interaction while introducing tools to appropriately critique what they observe and what they do.

## Take Home Messages

• Clinical communication curricula often utilise evidence from clinical settings to situate learning.

• Providing an introduction to understanding interaction at a foundational level may be useful, particularly for undergraduate students.
• Foundational learning on interaction can be informed by conversation analysis, with an emphasis on the ability to analyse talk.
• Integrating such learning can be done in an interactive and contextualised way.
• A strong foundation for understanding how interaction works is designed to support critical reflection on communication.

## Notes On Contributors

Sarah J White, PhD, is a Senior Lecturer in the Department of Biomedical Sciences, Faculty of Medicine and Health Sciences, Macquarie University. As a conversation analyst and qualitative health researcher, her work involves teaching and researching clinical communication and professional practice. https://researchers.mq.edu.au/en/persons/sarah-white
