# Supplementary material for: The science of talk in clinical science: using a conversation analytic approach as a foundation for communication skills training
Source: MedEdPublish (2016). 2020 Aug 28;8:224. Originally published 2019 Dec 9. [Version 2] doi: 10.15694/mep.2019.000224.2 (PMC10712510; doi:10.15694/mep.2019.000224.2)
Supplement: Peer review history

# Reviewer report: Reviewer response for version 2

**DOI:** 10.21956/mep.20256.r31431

**Published:** 2020-09-09

**Authors:** Ken Masters

**Affiliations:** 1 Sultan Qaboos University

This review has been migrated. The reviewer awarded 4 stars out of 5 I am pleased to see that the author has addressed the concerns raised in my review of Version 1. Although I would like to have seen a more explicit rounding of the results in the context of the Zone, the paper now has more clarity on the theoretical and practical context. As an aside: Rather than (or in addition to) citing Podolskiy’s definition and description as the source of the Zone, it probably would have been better to cite the primary source, Vygotsky, (whom Podolskiy acknowledges as having the ‘“absolute monopoly” on the notion of the zone of proximal development’))Nevertheless, a useful and enlightening read.

Reviewer Expertise:

NA

No decision status is available

# Reviewer report: Reviewer response for version 2

**DOI:** 10.21956/mep.20256.r31429

**Published:** 2020-09-06

**Authors:** Shamaila ijaz

**Affiliations:** 1 Lisca

This review has been migrated. The reviewer awarded 5 stars out of 5 The article describes a conversation analysis-informed curriculum for communication skills in an undergraduate pre-clinical science degree students. The abstract was more like a general writing. The introduction had detailed mergence with the topic at hand with updated references. The modules are described well in detail. The learning objectives has included curriculum from well recognized personalities. The future step is also good related to embedded communication in curriculum.

Reviewer Expertise:

NA

No decision status is available

# Reviewer report: Reviewer response for version 2

**DOI:** 10.21956/mep.20256.r31430

**Published:** 2020-08-31

**Authors:** P Ravi Shankar

**Affiliations:** 1 American International Medical University

This review has been migrated. The reviewer awarded 5 stars out of 5 I enjoyed reading the revised article. The additional information provided about the conversational analysis course is very helpful. The article will be of interest to a broad range of health science educators.

Reviewer Expertise:

NA

No decision status is available

# Reviewer report: Reviewer response for version 1

**DOI:** 10.21956/mep.19130.r27626

**Published:** 2020-03-19

**Authors:** Ken Masters

**Affiliations:** 1 Sultan Qaboos University

This review has been migrated. The reviewer awarded 3 stars out of 5 An interesting paper on using a conversation analytic approach as a foundation for communication skills training.The author begins by establishing the need for good communication skills and current training methods, particularly in the medical context. The paper then goes on to describe the various approaches taken to teaching communication skills.Overall, the paper is very clear in its approach, but there is a central point that is missing: the types of communication explored. There is a mention of non-verbal communication, but no real description of what is meant (one can assume body-language, but not much after that). For the rest of the communication scenarios, they appear (and I’m on dangerous ground making this assumption) to be face-to-face encounters only, and perhaps only one-on-one. Although videos are used in the teaching, there is no description of the use of video as a communication method (is this included in the non-verbal communication?). Most importantly, there is no mention of teaching written communication and electronic communication (written, video (recorded and live), audio, etc).It is possible that the author automatically includes all of these, but they do need to be identified more clearly; on the other hand, if the training is only on face-to-face verbal communication, in which non-verbal communication is mostly as a support to the verbal communication, then this should also be stated clearly.[As an aside, if electronic communication is not included in the current teaching, I would strongly urge the author to adjust the course so that it is included]The Abstract also refers to the zone of proximal development, but this does not appear to be discussed in the paper, nor are its theoretical underpinnings. It might be better to leave out the reference to it, or otherwise use it and the supporting theory in the paper.So, an interesting paper, but some clarity on the communication methods is required.

Reviewer Expertise:

NA

No decision status is available

# Reviewer report: Reviewer response for version 1

**DOI:** 10.21956/mep.19130.r27627

**Published:** 2019-12-10

**Authors:** P Ravi Shankar

**Affiliations:** 1 American International Medical University

This review has been migrated. The reviewer awarded 4 stars out of 5 This is an interesting paper on conversation analysis and communication skills for premedical students. One of the strengths is the interdisciplinary approach and the involvement of specialists from outside the traditional medical field. The approach has been well described and the module use material from a number of resources. It would be helpful if in the Introduction the author can provide more information about conversation analysis. Students are provided with opportunities to practice, analyze and reflect on their communication skills. I am not completely sure about the Bachelor of Clinical Science degree. I assume it is an undergraduate degree after which students can enroll for Medicine. The assessment integrates well with the curriculum and provides students with opportunities to observe and analyze their skills, reflect and improve.

Reviewer Expertise:

NA

No decision status is available
